# Soil-transmitted helminthiases among school-age children and their association with water, sanitation, and hygiene, Hawassa City, Southern Ethiopia

**DOI:** 10.1371/journal.pntd.0011484

**Published:** 2023-07-28

**Authors:** Belachew Bokicho, Dejene Hailu, Bethlehem Eshetu, Male Matie, Tafese Tadele

**Affiliations:** 1 Neglected Tropical Diseases Team, SNNP Regional Health Bureau, Hawassa, Sidama, Ethiopia; 2 School of Public Health, College of Medicine and Health Sciences, Hawassa University, Hawassa, Sidama, Ethiopia; 3 Disease Prevention and Health Promotion Directorate, SNNP Regional Health Bureau, Hawassa, Sidama, Ethiopia; Federal University of Agriculture Abeokuta, NIGERIA

## Abstract

**Background:**

Soil-transmitted helminthes pose the main health impact in tropical and sub-tropical regions, with children being at increased risk of infection. This study assessed the prevalence of soil transmitted helminthes among school children and their association with water, sanitation, and hygiene condition in Hawassa City, southern Ethiopia.

**Methodology/Principal findings:**

A cross-sectional study design was employed on randomly selected 549 school-age children from 11 schools by using a multistage sampling method. Data were collected using a structured questionnaire and observation checklist. Stool samples were collected and tested as fresh within 2 hours using the Kato-Katz technique as standard procedure. Data were analyzed by SPSS software; results were summarized using descriptive statistics, and a logistic regression model. Levels of considerable tests were determined with a 95% confidence interval and P-values <0.05.

The overall prevalence of soil-transmitted helminthes was 49.7% (95% CI: 45.7%, 53.9%). Overall, water and latrines services were below the standard of 20 liters per person per day and one latrine seat per 50 boys and 25 girls respectively. In particular, no habit of washing hands with water and soap, 1.9%, (95% CI: 1.2%, 3.0%); inaccessible to safe drinking water, 10.8%, (95% CI: 3.96%, 30.26%); inaccessible to improved latrine, 10.8%, (95% CI: 1.5%, 78.4%); and practicing open defecation at school compound, 9.4%, (95% CI: 1.5%, 57.2%) were the main issues of concern observed.

**Conclusions/Significance:**

Almost half of the studied children were infected with one or more soil-transmitted helminthes. Schools had inadequate water, sanitation, and poor personal hygiene practices. The infection by soil-transmitted helminthes among school children was high. This study has indicated that water, sanitation, and hygiene-related factors were the main risk factors for helminthes infestation in the study area. The school community needs to focus on actions that promote hygiene practices in the school.

## Author summary

This study has appreciably indicated that the prevalence of soil-transmitted helminthes are association with water, sanitation, and hygiene factors that were mainly affected the school-age children as they are a highly vulnerable group of the community especially within the city setups. This is valuable to health and education sectors to direct planning and resources to target the common health problems relating to the soil-transmitted helminthes and water, sanitation, and hygiene factors. It also gives insights about the current situations of the school environment as well as corrective interventions that will be implemented for children infected with STH. Further, the finding contributes to strengthening health-promoting behaviors.

## Introduction

Soil-transmitted helminthes (STH) and lack of safe Water, Sanitation, and Hygiene (WASH) are important challenges to global development. Soil-transmitted helminthes are the most common and persistent parasitic infections, particularly in tropical and sub-tropical regions of the world. Although it is prevalent among all age groups, World Health Organization (WHO) reports that school-aged children (SAC) are at higher risk of infection by STH [1]. The previous systematic review and meta-analysis show that access to adequate safe water, improved sanitation facilities, and good personal hygiene is associated with 33%–70% lower odds of STH infection [2].

The high prevalence rate of STH is attributed largely to socio-economic status, poor sanitation, inadequate medical care, and absence of safe drinking water. The STH infections are mainly associated with children’s habits of playing in dirty places or handling of infected soils, eating with soiled hands, unhygienic toilet practices, and drinking or eating contaminated water and food respectively [3].

The current global estimates indicate that 4.5 billion individuals are at risk of STH infections and they cause clinical morbidity in approximately 450 million people. They are one of the world’s most important causes of physical and cognitive growth retardation in children leading to attention deficits, learning disabilities, school absenteeism, higher dropout rates, and lower wage-earning capacity in adulthood, and lower Gross Domestic Product (GDP) for the nation [4]. Soil-transmitted helminthes infections are commonly distributed in tropical and subtropical areas, with the highest proportion occurring in sub-Saharan Africa, the Americas, China, and East Asia [5].

In Ethiopia, previous studies have shown that the STH infections prevalence was high in the lower altitudes, *Ascaris lumbricoides* being the majority in different communities, usually occurring together with either *Trichuris trichuria* and/or hookworm. This is mostly attributed to Ethiopia’s lowest quality of safe water and improved latrine coverage. A three-year longitudinal study on integrated school health and nutrition approaches in 30 government primary schools in southern Ethiopia reported a strong association between sanitation, hookworm infection, anemia, stunting, and wasting [6].

Further studies conducted in Arbamich town in southern Ethiopia reported 27.5% of STH prevalence as well as their determinant factors among SAC [5]. In Butajara town, Southern Ethiopia, 23.3% of pre-school age children (PSAC) were infected with one or more STH species [1].

This study was conducted to estimate STH prevalence among SAC and their association with WASH factors in Hawassa City, southern Ethiopia. Recommendations are provided for further interventions.

## Methods

### Ethics statement

An ethical clearance was sought from Hawassa University’s Institutional Review Board (IRB) and an approval letter (Ref. No: IRB/087/11) was obtained. All ethical principles (respect for persons, beneficence, and justice) were considered while conducting the study.

All the study participants were informed about the benefits and risk of this the study through a written consent and information sheet. The written consent from schools’ principals to conduct the interview and stool examination of SAC based on their willingness was obtained. The written consent was obtained from the parents of the children for their participation in the study. The verbal assent of children was obtained prior to data collection and the data collection processes and stool sampling was done based on voluntary participation.

Coding of each subject was done to maintain the confidentiality of the information. Finally; participants with positive test results were referred to Hawassa City Health Department for treatment.

A school-based cross-sectional study design was employed to estimate STH prevalence and its association with WASH status. Eleven government-owned primary schools were randomly selected out of the existing 42 schools (hosting 5,426 students) found in Hawassa City Administration and involved in the study were pupils attending grades 1–8. The mass drug administration (MDA) using a single dose of albendazole 400mg was carried out by the NTD program of the Hawassa City Administration Health Department six months before the commencement of the study. During the recent MDA 57% therapeutic coverage was reported.

The sample size estimation was determined by considering STH prevalence an anticipated population proportion of 54%, obtained from a similar study conducted among SAC [7] in Jimma Town, South West Ethiopia, and 95% level of confidence, 5% marginal error, and 10% non-response rate. We considered a design effect of 1.35 to account for sampling error [8]. That yielded a minimum sample size of 567 students. The number of study participant students from those selected schools was proportionally allocated based on the total number in each school.

A multistage sampling method was conducted to select the study units. In the first stage, 11 government primary schools were selected out of 42 schools. Next, grades and sections were randomly selected. Systematic random sampling was employed to access individual students through the class roster. The first student was selected arbitrarily from each selected section. Then, the selection continued using the pre-calculated sampling interval till the numbers to be sampled from the respective classes were achieved [7].

The data were collected by using a structured questionnaire, observations checklist, and laboratory diagnosis. Personal hygiene status focusing on cleanliness of fingers and fingernails were observed using the checklist. Data on school WASH conditions and environmental setup was done through an interviewer-administered questionnaire and children’s basic hygienic practices were assessed prior to the laboratory test by using an observation checklist for children whose fingers were clean and whose nails were cut short and other hygienic practices.

Stool samples were collected from the schoolchildren in sterile containers, which were labeled with the participant’s unique identifier. Stool samples collected from the school children in sterile containers were transported to the laboratory for analysis with the help of trained laboratory technicians.

Specimens were processed within 2 hours of collection for microscopic examination of STH species by using the Kato-Katz technique as standard procedure [9]. A portion of the sample was processed by the Kato-Katz method using a template holding 41.7mg of stool, two slides per stool [10]. The number of parasites’ eggs was counted and multiplied by 24 to obtain the number of parasites per gram of stool [4]. The infection intensities were recorded and graded as light, moderate, or heavy by using the number of eggs per gram (EPG) of stool according to World Health Organization (WHO) thresholds [11].

Statistical Package for the Social Sciences (SPSS) statistical software version 20 was used for data entering, cleaning, and analysis. The association between STH and WASH was decided by using logistic regression. Variables for the final model were selected at p-value bivariate <0.20 and the association was declared from multivariable models with p-value <0.05.

## Results

### Socio-demographic characteristics

Among 567 students targeted in this study, a total of 549 participants were tested for STH infection as well as their hygienic conditions were assessed at 11 selected government primary schools in Hawassa city with an overall response rate of 96.8%. Out of those children, 220 (40.1%) fell in the age range of 5–9 years, and 324 (59.9%) in the age range of 10–14 years. 284 (51.7%) were male, 402 (73.2%) were in grades 1–4, and 147 (26.8%) were in grades 5–8 “Table 1”.

**Table 1:** Socio-demographic characteristics among School age-children in Hawassa city, southern Ethiopia.

Variable (N* = 549)	Number of children Sampled	Percent %
**Age range**		
5–9	220	40.1
10–14	324	59.9
**Grades**		
1–4	402	73.2
5–8	147	26.8
**Sex**		
Male	284	51.7
Female	265	48.7

*—N: Total number of the study participants

### Prevalence of soil-transmitted helminthes

Of 549 children tested, the overall prevalence rate of STH was 273 (49.7%, 95% CI: 45.7%, 53.9%). A high prevalence (over 50%) was recorded among children 5–9 years old, 116 (52.7%), among males, 142 (51.1%) and 188 (49.6%) among those in grades 1–4. The three commonest species recorded were *A*. *lumbricoides* 232 (42.3%), *T*. *trichuria*, 108 (19.7%), and Hookworm 20 (3.6%). Most were light intensity infections (29.3%), some were moderate (9.3%) and a few heavy (3.6%) infections of *A*. *lumbriciodes* “Table 2”.

**Table 2:** The STH infection and intensity level of each species among School age-children in Hawassa city, southern Ethiopia.

Variables	Intensity (EPG*)	Frequency	Percent %	95% CI	
				Lower	Upper
*A*. *lumbricoides*	Light (1–4,999)	161	29.3	25.5	33.6
	Moderate (5000–49,999)	51	9.3	7.1	11.8
	Heavy (> = 50,000)	20	3.6	2.2	5.1
	**Sub-total**	**232**	**42.3**	**37.9**	**46.8**
*T*. *Trichiura*	Light (1–999)	105	19.2	15.8	22.6
	Moderate (1000–9,999)	3	0.5	0.0	1.3
	**Sub-total**	**108**	**19.7**	**16.2**	**23.3**
Hookworms	Light (1–1,999)	20	3.6	2.0	5.1
**Any STH**		**273**	**49.7**	**45.7**	**53.9**

*—EPG: Eggs per Gram

**Fig 1:**
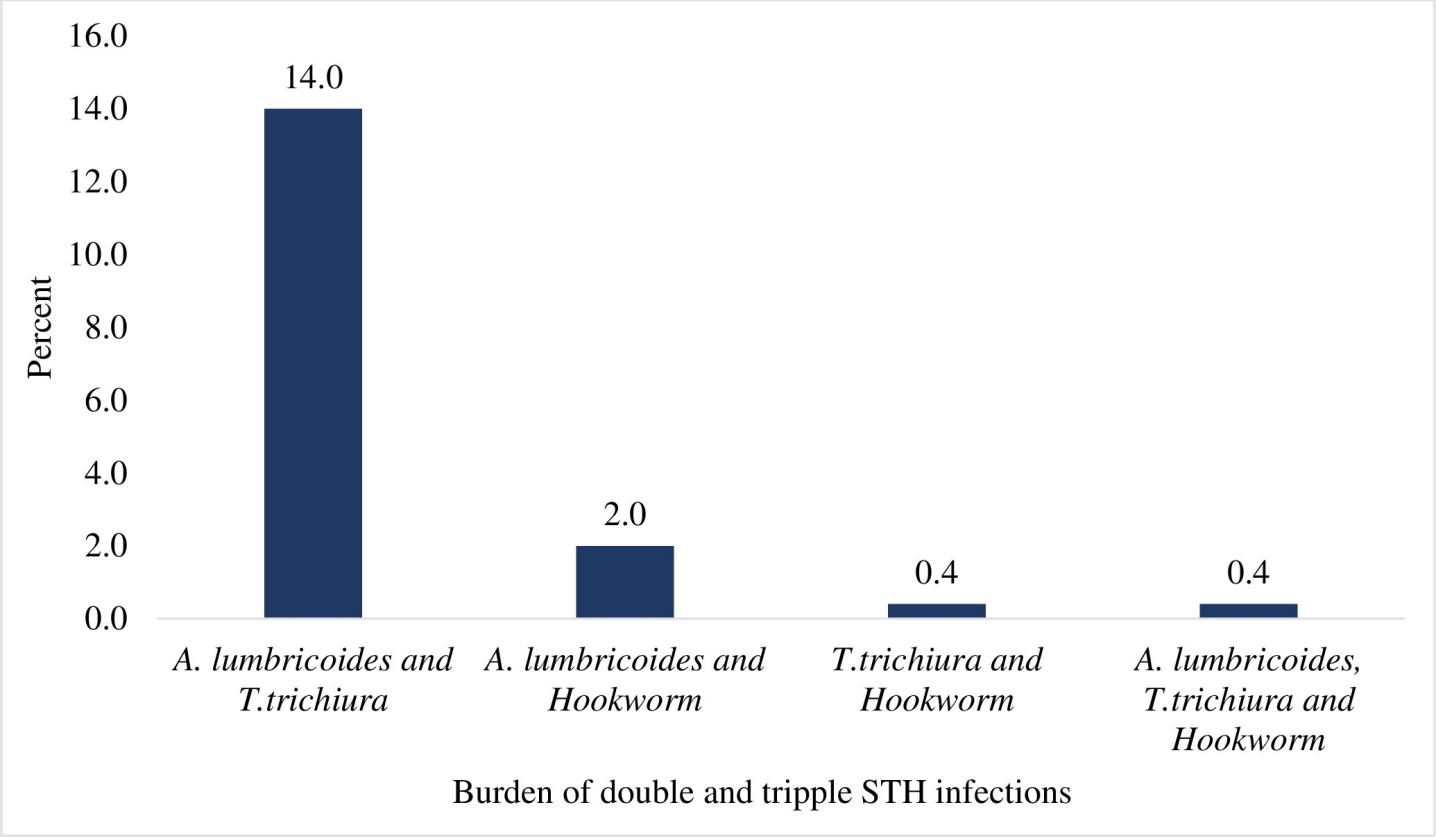
Burden of double and triple infection of STH species among SAC.

The study found double infections, 87 (16.2%), and triple infection, 2 (0.4%). *A*. *lumbricoides* and *T*. *trichuria* infection was recorded for 76 (13.8%), A. lumbricoides and Hookworm infections: 11 (2%), and *T*. *trichiuria* and Hookworm infections: 2 (0.4%). A small proportion of triple infections of *A*. *lumbricoides*, *T*. *trichiuria* and Hookworm were also seen: 2 (0.4%) children (Fig 1).

### Water supply and sanitation facilities status

In this study, 10 (90.9%) schools had access to water supply from taps located in the school compound, and 4 (40%) had access to tanker/reservoir in their school compounds. However, in all schools, the water supply availability was intermittent (not available at all times) in other words the water supply system was inadequate as the standard of 5 liters per person per day for all SAC and staff [12]. The amount of water supplied in the studied schools on average was only 1.3 liters per person per day.

All schools had access to latrines, of which, 6 (54.5%) had a water carriage system, 5 (45.5%) had a simple dry pit latrine while 1 (9.1%) of schools had access to a ventilated improved pit latrine. Nine of the 11 schools (81.9%) schools had separate latrine blocks for students but in all schools, the latrine seats were inadequate compared to the standard of one latrine seat for 50 boys and 25 girls [13]. Overall, 160 boys and 140 girls served in one seat of a latrine in their separate blocks, respectively. Concerning hygiene facilities, 7 (63.6%) of school latrines had hand washing facilities within nearby distance with water and soap for hand washing while some had no soap “Table 3”.

**Table 3:** Water supply and sanitation facilities status in the sampled schools.

Variables	Yes		No	
	Frequency	Percent %	Frequency	Percent %
School has access to safe water supply	10	90.9	1	9.1
Water access from Tanker/reservoir (Rotto)	4	40.0	6	60.0
Water directly fetching from taped pipes for drinking (has no storage)	3	27.3	8	72.7
Methods of water drawing from containers through dipping	3	27.3	8	72.7
Methods of water drawing from containers through pouring	8	72.7	3	27.3
Schools had water treatment by the municipality	2	18.2	9	81.8
Schools had water carriage systems of latrine	6	54.5	5	45.5
Schools had a simple dry pit latrine	5	45.5	6	54.5
Schools had ventilated improved pit latrines	1	9.1	10	91.9
Schools had separate latrine blocks for male and female students	9	81.8	2	18.2
Schools had urinals around the latrines	8	72.7	3	27.3
Schools had hand-washing facilities nearby the latrines	7	63.6	4	36.4
Had School WASH club	2	18.2	9	81.8
Conducting sanitation campaign	8	72.7	3	27.3

Knowledge of the principals about STH affection and its association with WASH factors

The study revealed that the entire studied schools conducted de-worming program for preventing STH, and the principals had information about STH infection. Similarly, all principals reported to have known about STH and their association with WASH factors. Interestingly among them, 9 (81.8%) mentioned ascariasis and 6 (54.5%) hookworm but all had no information about trichuriasis. The majority, 9 (81.8%) principals stated that children under 5 years were more affected by STH infection, while only 2 (18.2%) mentioned that children 5–14 years were at risk of STH infection.

**Fig 2:**
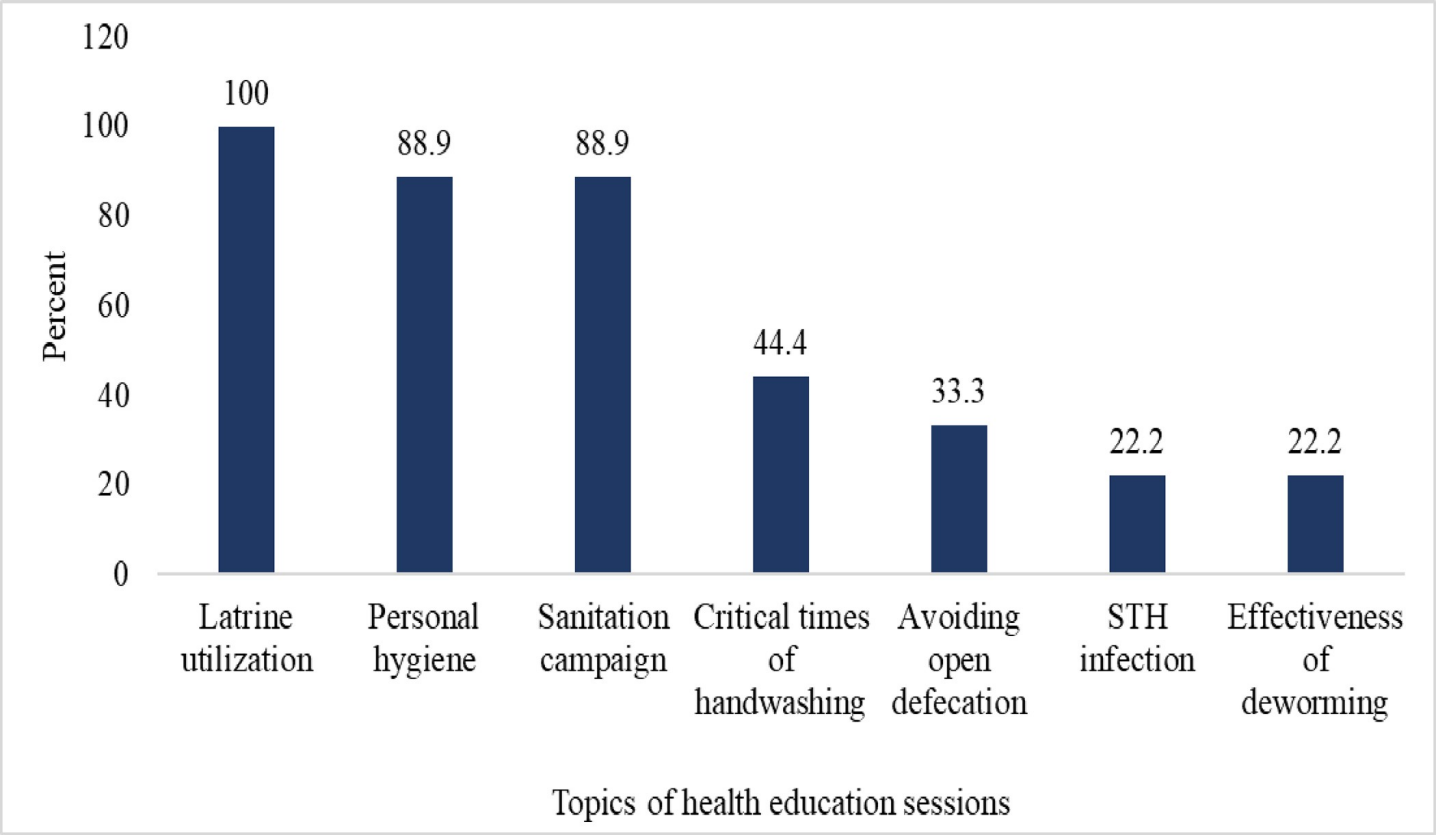
Topics of health education sessions in study schools in Hawassa City, southern Ethiopia.

All respondents/principals in the studied schools had information about WASH and about 9 (81.9%) of the schools had hygiene education sessions and all of them included a proper utilization of latrine in their hygiene education sessions. But only 2 (18.0%) of the schools included a problem of STH infection in their hygiene education sessions. About 8 (72.0%) of the schools had sessions on sanitation campaigns while 5 (45.0%) held sessions on critical hand washing times (Fig 2).

### Hygienic behavior and practice of school-age children (SAC)

All SAC were assessed for their hygiene behavior during the collection of stool samples. Children who wore shoes, 531 (96.7%); those who had access to functional drinking water from safe sources at home (piped, protected well, and treated) were 497 (90.7%), children who had access to an improved latrine at home (VIP, water carriage and pour flash), were 256 (46.6%). Children whose fingers were clean and nails cut short were 245 (44.6%). In addition, a few proportions of the children 99 (18%), and 22 (4%) had a habit of swimming in stagnant water and practicing open defecation around the home respectively “Table 4”.

**Table 4:** Hygienic behavior of the school-age children in Hawassa City, southern Ethiopia.

Variable (N* = 549)	Yes		No	
	Frequency	Percent %	Frequency	Percent %
Children whose fingers were clean and nails cut short	245	44.6	304	55.4
Children with the habit of swimming in stagnant water	99	18.0	450	82.0
Children who wore shoes	531	96.7	18	3.3
Children with access to safe drinking water	497	90.5	52	9.5
Children who access improved latrines at home	256	46.6	293	53.4
Children who practice open defecation around home	22	4.0	527	96.0

*—N: Total number of the study participants

Overall, 495 (90.2%) children practiced hand washing before meal and 372 (67.8%) after using the latrine. Of those who practiced hand-washing only 178 (32.4%) used clean water and soap.

### Water, sanitation and hygiene factors association with soil-transmitted helminthes

The prevalence of STH among the SAC in the studied government primary schools in Hawassa City were analyzed for association with WASH factors and described by using bivariate and multivariate analysis.

In the bivariate analysis factors that had been found to have an association and were potential candidates for multivariate analysis. These included: children’s finger and nail cleaning status, children’s hand washing practice and use of clean water and soap for hand washing, children’s access to safe drinking water and access to an improved latrine, and children’s practice of open defecation around the home. Also based on the school environment and facilities of those children found; sanitation campaigns, hygiene education sessions, and the latrine cleanliness were independently associated with STH, taken as a p-value < 0.20. And they were candidates for multivariate analysis; statistically considerable recruited and fitted the model properly.

The potential predictor variables for the prevalence of STH as identified by multivariate logistic regression analysis included: the cleanliness status of children’s fingers and nails, children’s use of clean water and soap for hand washing, access to clean drinking water at home, access to an improved latrine at home, the practice of open defecation. Furthermore, sanitation campaign conducted at schools and the cleanliness status of school latrines were considerably associated with infection of STH by using p-valve < 0.05 as shown in “Table 5”.

**Table 5:** Bivariate and multivariate logistic regression analysis of WASH factors associated with STH among SAC in Hawassa City, southern Ethiopia.

Variables		STH Status		COR (95% CI)	AOR (95% CI)
		Positive	Negative		
Fingernails clean	Yes	84	161	1.0	1.0
	No	189	115	3.15 (2.21, 4.47)	3.48 (2.31, 5.24)
Wash hands after using latrines	Yes	161	211	1.0	1.0
	No	112	65	2.26 (1.56, 3.26)	1.36 (0.82, 2.24)
Use clean water and soap for hand washing	Yes	56	122	1.0	1.0
	No	217	154	3.07 (2.10, 4.48)	1.93 (1.23, 3.03)
Had access to safe drinking water sources at home	Yes	226	271	1.0	1.0
	No	47	5	11.27 (4.41, 28.82)	10.94 (3.96,30.26)
Had access to an improved latrine at home	Yes	257	269	1.0	1.0
	No	16	7	2.39 (0.97, 5.91)	10.83 (1.49, 78.43)
Practiced open defecation around home	Yes	19	7	2.875 (1.19, 6.95)	9.36 (1.53, 57.21)
	No	254	269	1.0	1.0
Conduct sanitation campaigns at Schools	Yes	72	110	1.0	1.0
	No	201	166	1.85 (1.28, 2.66)	2.12 (1.13, 3.97)
Has hygiene education sessions at schools	Yes	26	50	1	1
	No	247	226	2.10 (1.27, 3.49)	2.26 (0.71, 7.22)
Schools have access to a water supply	Yes	222	262	1.0	1.0
	No	51	14	4.29 (2.32, 7.97)	3.78 (0.93, 15.28)
Schools have access to the simple dry pit latrine	Yes	112	163	1.0	1.0
	No	161	113	2.07 (1.48, 2.91)	1.55 (0.80, 3.01)
School latrines cleaned appropriately	Yes	243	265	1.0	1.0
	No	30	11	2.974 (1.46, 6.06)	3.24 (1.39, 7.54)

## Discussion

The overall prevalence of STH was 49.7% among SAC and they were affected by single or multiple STH species mostly of light or moderate intensity. The schools’ WASH facilities were inadequate for SAC, based on the standards of water supply system in schools 5 liters per person per day for all SAC and staff [12] and one latrine seat for 50 boys and 25 girls [13]. Most of the children have had also an improper practice of hygienic behaviors and higher than half of them did not use clean water and soap when they washed their hands. These WASH factors were considerably associated with the STH infection was found in the finding and the results are aligned with previous studies [1,7,14–17].

In Ethiopia, a cross-sectional study showed an overall prevalence of 53.5% of STH infections, and a prevalence of 57% was reported among students aged 11–15 years in government primary schools in Jimma town [7]. In another study in Durbete town, North West Ethiopia, a prevalence of 54.9% STH infection among SAC, was reported [14].

Despite the fact that, many African countries have made significant achievements in reducing STH, it remains high in some countries such as Kenya. For instance, a prevalence of 40.7%; 22.7%; and 28.8% STH was reported for *A*. *lumbricoides*, and *T*. *trichuria*, and hookworm respectively in Nairobi, Kenya. A prevalence ranging from 22% to 71% between sub-village sectors of STH was reported among SAC and had at least a single STH infection [15].

A high proportion of schools have access to water while all had latrines, but the water supply system and latrine facilities in all schools were inadequate as a minimum of 5 liters per person per day of water supply in schools [12]. In this study, most of the children had improper hygiene practice behaviors (unclean hands and fingers nails were not cut short). More than half of the children did not use clean water and soap when they washed out their hands and they also practiced open defecation around their homes. The level of hygiene facilities and hygiene behavior of children found in this study are comparable with previous studies [7,14].

According to Ethiopia’s Ministry of Education, School WASH facilities mapping, 2017, in the region, the proportion of primary schools with access to an improved water supply facilities were 30.6%, access to improved latrine, 36%, access to inclusive improved latrine with hand washing facilities, 33.6%, and latrine stance to student ratio is 1:222 and only 1.8% full WASH facilities [16]. This study demonstrated that over two-thirds of the respondents did not have access to improved WASH services and these factors have contributed to that STH infection prevalence being reported as high in Durbete town, northwestern Ethiopia [14].

Lack of access to improved WASH in schools such as Kenya (17) and Tanzania [18] adversely affects students in several ways. In schools with inadequate WASH facilities, students, especially girls who need adequate water supply at schools, particularly for menstrual hygiene have no options other than staying at home during these periods. This severely will result in poor academic performance of girls as they repeatedly miss classes. Moreover, unclean school latrines could serve as the main sources of excreta-borne infections affecting their health.

These findings are consistent with previous studies. In Butajira, southern Ethiopia, higher STH infection prevalence was observed in children with untrimmed fingernails, AOR = 3.2 (95%CI: 1.8–5.5) compared to those with trimmed fingernails [1]. Similarly, a pervious study which was conducted in Duberty town, northwest Ethiopia, reported that children who did not have the habit of washing their hands before eating had 3.80 (95% CI: 1.02, 14.23) higher odds of being infected with STH than children who had practicing washing their hands before eating [14].

In Kenya, evidence shows that the STH infection rate was lower among children who had access to treated drinking water in their households compared to those living in households where drinking water was not always treated exhibiting STH prevalence rate of 81% (95% CI: 66%-99%) [15].

These findings generally imply that there is still a big gap in schools to achieve the minimum requirement for access to basic WASH services in some developing countries. Some variations observed between the current and previous studies are attributed to variations in socio-demographic status, behaviors pertinent to personal hygiene, and the habit of shoe wearing, water handling at home and environmental sanitation of the school compound.

In addition to this, waste disposal pit within the school, type of latrine accessible at home and at schools, their cleanness, and sufficient number to children which have a great impact on the distribution of STH have been considered [7].

These findings were shown that most of the schools have not improved WASH facilities and weak health promotion and poor personal hygiene practice behavior of the students’ have caused for this a high level of STH infection. Thus, improving the availability of clean and sufficient toilet facilities, safe and adequate water supply, and improved environmental sanitation of the schools compound and health education promotion on personal hygiene practices of the students will be needed highly needed to overcome these STH problem. Furthermore, frequent assessment and appropriate intervention of those de-worming programs as well as maintaining WASH services in the schools are very crucial to overcome STH infections and it should be an integral part of the control and prevention strategies.

### Limitations and strengths of the study

This study, being a cross-sectional survey, is subject to some limitations, the information generated through interviewer-administered interviews is not free of recall bias. Social desirability bias and egg or chicken dilemma is also likely limitation that might affect the reliability of the findings. Some number of resistances and missing stool sampling in SAC, especially among the age group 10–14 years and grade level 5-8^th^ was recorded as 18 (3.2%).

The strengths of this study were a good opportunity to indicate that the STH prevalence and WASH factors are association within city schools yet did not study before in the study area. The data collection and examination of stool samples which is reliable and increase the value of the study finding. It has been processed by using the Kato-Katz technique as standard procedure [9] through trained laboratory technicians and experienced health professionals. And also, the good commitments of school principals and children’s good willing have greatly contributed to successfully completion of this finding.

## Conclusion

In general, the STH prevalence observed in this study was high as almost half of SAC were infected with at least a single or more STH species. Schools with less or unimproved WASH facilities as well as children in schools who had poor practices of personal hygiene were more likely to factorized for this infection. And also it was considered that real confounding WASH factors were associated with a high extent of STH infection in these study findings.

This high prevalence of STH infection calls for a periodic de-worming to reduce transmission, morbidity, worm burden, and other related health problems among SAC. The health and education sectors are implementing improved WASH services such as safe water supply system and environmental sanitation measures as well as appropriate health education in the schools should be highly considered.

In addition to that, further study through considering with large sample size including the private schools will be needed as independently to investigate the STH risk factors among SAC in the city. And also proper interventions towards to overcoming the risk of those findings are mandatory.

## Supporting information

S1 Data Data used for the study. (SAV)
